# Successful Pregnancy Using the NxStage Home Hemodialysis System

**DOI:** 10.1155/2016/1358625

**Published:** 2016-02-02

**Authors:** Yasmin Brahmbhatt, Arinze Ikeme, Navjyot Bhogal, Vincenzo Berghella

**Affiliations:** ^1^Division of Nephrology, Thomas Jefferson University, 833 Chestnut Street, Suite 700, Philadelphia, PA 19107, USA; ^2^Department of Obstetrics and Gynecology, Thomas Jefferson University, 833 Chestnut Street, 1st Floor, Philadelphia, PA 19107, USA

## Abstract

Pregnancy in the setting of the uremic milieu of renal disease has a lower success rate than in the normal population and is a rare event. While intensified renal replacement therapy (RRT) during pregnancy can lead to improved outcomes, most studies have focused on nocturnal hemodialysis as the main RRT in pregnancy. Although thousands of patients use the home NxStage System One short daily hemodialysis (SDHD) machine in the United States, pregnancy outcomes with this therapy are unknown. The NxStage System One uses low-volume dialysate and hence small and middle molecule clearance may differ compared to conventional therapies and affect pregnancy outcomes. We report a case of a successful conception and pregnancy using the home NxStage system. The NxStage system may provide an alternative to the more routinely used NHD or standard SDHD therapies for women of childbearing age.

## 1. Introduction

Pregnant women with ESRD are exposed to a range of potential hazards including accelerated hypertension, intrauterine growth retardation (IUGR), polyhydramnios, preterm birth, and low birth weight [1].

Nocturnal hemodialysis (NHD) has been studied in this population and has been shown to improve pregnancy outcomes, likely due to the maternal host environment becoming more hospitable for successful pregnancy in the setting of augmented uremic clearance [2]. A recent cohort comparison between the Toronto Pregnancy and Kidney Disease Registry for Pregnancy in Dialysis Patients and the American Registry for Pregnancy in Dialysis Patients revealed a dose response between dialysis intensity and pregnancy outcomes, with live birth rates of 48% in women dialyzed less than 20 hours/per week and 85% in women dialyzed more than 36 hours per week, with a longer gestational age (37 weeks in Canadian group versus 27 weeks in the American group) and greater infant birth weight for women dialyzed more intensively [3]. Retrospective data suggests that maintaining a predialysis BUN of ≤50 mg/dL leads to longer gestation and increased likelihood of a successful pregnancy [4]. Frequent hemodialysis also improves fluid management with less hypotension or hypertension, reduced inflammation, and improvement in endothelial function [4]. The positive relationship between intensified dialysis and improved maternal and fetal outcomes has led to increased dialysis delivery becoming the standard practice in pregnant patients [5]. However, there has been little research into whether the home NxStage System One can provide similar outcomes in this population. The differing dialysis modality is important to note as the NxStage system uses low-volume dialysate and hence small and middle molecule clearance may differ and affect pregnancy outcomes (Table 1).

In fact, there is little data on solute clearance with the NxStage System One. One study looked at 5 patients on the NxStage System One and measured B2 microglobulin, phosphorus, urea, and potassium clearance [6]. It found favorable B2 microglobulin clearance (640 mg/week) when compared to conventional IHD (127 ± 48 mg/wk) and nocturnal hemodialysis (585 ± 309 mg/wk). Phosphorus removal was 4.16 grams per week compared to other studies reporting phosphorus clearance of 2.74–2.79 grams/week with CAPD and 2.36 grams/week with conventional hemodialysis. Standard Kt/V averaged 2.5 ± 0.3 per week and correlated with the dialysate-based weekly Kt/V. Ultrafiltration volumes were 10.5 L per week in this study which could greatly increase the convective clearance of small and middle molecules and may not accurately reflect clearances in a pregnant ESRD patient where ultrafiltration volumes may be much less.

Interestingly, a recent study by Weinhandl et al. [7] compared matched home hemodialysis (all using NxStage System One) with peritoneal dialysis patients. The data suggests that, relative to PD, daily HHD was associated with decreased mortality, hospitalization, and technique failure. The absolute rates of cardiovascular mortality and infection related mortality were significantly less in the HHD group. This was an observational study. Differences in biochemical parameters, dialysis frequency, duration of treatments, and residual renal function were beyond the scope of this paper. Despite the known low-volume dialysate and shorter therapy time (compared with PD), the differences in outcomes were striking.

We report a case of successful conception and pregnancy using the home NxStage System One as we believe this is a unique case due to preserved fertility, conception, and successful pregnancy occurring in a patient with longstanding ESRD on low-volume dialysate-based renal replacement therapy.

## 2. Case Report

A 29-year-old obese African American female with ESRD from biopsy proven idiopathic collapsing focal segmental glomerulosclerosis initiated SDHD with the NxStage System One. Her blood pressure was in the range of 120–130/75–85 mmHg and she was clinically euvolemic. She had 300 mL residual urine output. She underwent two-hour sessions of HD five days per week via a brachiocephalic arteriovenous fistula. Her initial dialysis prescription included 30 L dialysate and a flow fraction (dialysis flow rate, *Q*_*d*_/blood flow rate, *Q*_*b*_) of 40%. Weekly Kt/V was 2.35, with average ultrafiltration of 2.5 L/session. She had regular thirty-day menstrual cycles on this prescription.

Three years later, in 2012, she developed amenorrhea. Pelvic ultrasound confirmed a pregnancy at 8 weeks' gestation. She underwent multidisciplinary care with close obstetric and nephrology observation with monitoring of weight gain, evaluation of dry weight based on symptoms, examination, vital signs and interdialytic weight gain, and laboratory tests for preeclampsia twice a month. Prenatal vitamins and folic acid 1 mg daily were prescribed. Fetal growth monitoring scans at monthly prenatal visits from the 8th week to the 14th week were normal. Average systolic BP continued to range from 120 to 130 mmHg. Her urine volume gradually increased to 1300 mL/day. The dialysis prescription was subsequently intensified, with a target predialysis BUN < 45 mg/dL (Table 2).

Hemodialysis time was increased to 21 hours per week by 16 weeks of gestation. Due to her increased urine output, conservative ultrafiltration was adopted with no more than 0.5 L per session. Erythropoietin and intravenous iron were prescribed to target hemoglobin of 10.5 mg/dL. Protein supplementation was initiated to aim at a serum albumin 4 gm/dL.

**Figure 1:**
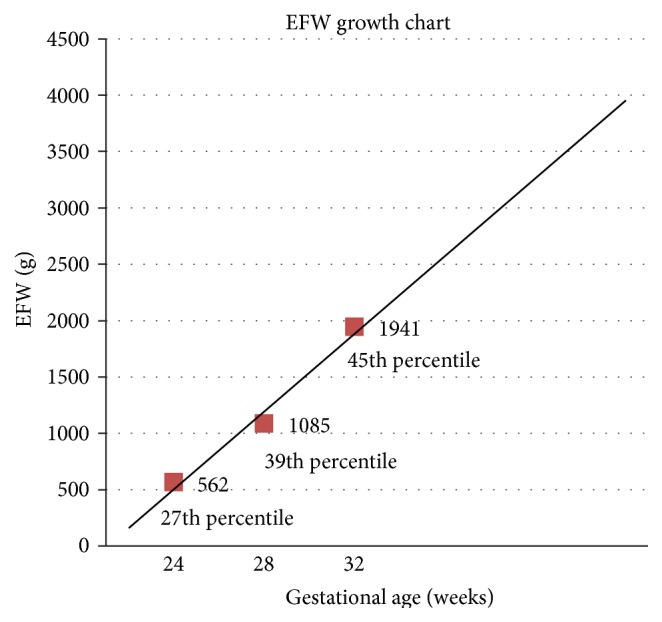
EFW: Estimated Fetal Weight.

The patient had prenatal care visits at least every 4 weeks and more frequently after 26 weeks. Beginning at 18 weeks' gestation, fetal growth, amniotic fluid volume, and placental integrity were monitored with pelvic ultrasound every two weeks in addition to twice weekly nonstress tests (NST) for assessment of fetal activity and well-being. These studies were within normal limits. As fetal growth was normal, uteroplacental blood flow was not evaluated as it was assumed to be normal (Figure 1).

By 20 weeks' gestation, our patient was 7 kg above her prepregnancy estimated dry weight, which was attributed to physiologic weight gain in pregnancy. By 28 weeks, she underwent 26 hours of hemodialysis per week. Elevated systolic BP of 150–160 mmHg was observed at this time, with no clinical evidence of volume overload. Preeclampsia was suspected, although aggressive blood pressure control was not pursued due to symptoms of nausea and dizziness with systolic blood pressure less than 130 mmHg during hemodialysis treatments.

She was subsequently admitted to the hospital at 33 weeks' gestation due to worsening hypertension (SBP, 170 s mmHg) and elevated liver enzymes. Daily hemodialysis was conducted with no ultrafiltration. Magnesium sulfate was prescribed for eclampsia prophylaxis. Labor was induced at 33 weeks' gestation, and a male infant weighing 1.93 kg was delivered vaginally with Apgar scores of 8 and 8 at 1 and 5 minutes, respectively. The infant was monitored in the neonatal care unit for 2 weeks and both mother and infant were subsequently discharged. Both were doing well at their 6th-week postnatal visit. The child is now almost 3 years old, doing well.

## 3. Discussion

Home hemodialysis (HHD) was popular before the onset of the Medicare ESRD program in the 1970s. Subsequently HHD declined significantly but has recently become more popular. Forty-nine thousand prevalent dialysis patients received RRT at home in 2012 [8]. Of these patients, 16.3% were treated with hemodialysis and 83.7% were treated with peritoneal dialysis (PD). Overall, HHD was 63% higher in 2012 than in 2002. Data on how many females of childbearing age on HHD are using the NxStage System One is not readily available. However, as of February 2011, 5000 patients in the United States were using NxStage System One, of which 36% were female (versus 45% female on IHD). Twenty-five percent of all patients on NxStage One are under the age of 45 years (data reproduced with permission from NxStage). This means that, as of 2011, approximately 62% of all home hemodialysis patients were using the NxStage System One. Generally, patients who are candidates for home hemodialysis have (1) motivation and are physically able to do treatments at home, (2) arteriovenous access, (3) compliance with treatments, and (4) a caregiver present during the treatment in case of emergency. General contraindications are (1) living alone, (2) being homeless, (3) nonadherence to therapy, and (4) dialysis catheter as hemodialysis access.

The NxStage One machine may provide lower Kt/V (due to low dialysate volumes) than conventional machines and its use in pregnancy may raise concerns. The patient was reluctant to move to in-center hemodialysis or nocturnal hemodialysis due to psychosocial reasons. She was very motivated to remain on the NxStage machine. We increased the dialysis duration to the maximum she was able to tolerate (26 hours) and increased the dialysate volume from 30 L to 45 L to achieve a target predialysis BUN of less than 50. The dialysis time rose by 160% but the dialysate volume only increased by 50%. Kt/V only increased by 50% as well. These changes increased the middle molecule clearance more than small molecule clearance. The improved residual renal function had a significant effect on both types of molecules but the middle molecule clearance is more striking. Despite the lower clearance using the NxStage machine, increasing the dialysis time without proportionate increase in dialysate volume may provide a better clinical effect than the Std. Kt/V indicates. It is possible that the increased dialysis time may provide increased middle molecule clearance and may be more important in pregnancy and deserves further study.

The NxStage system uses a lactate based dialysate as opposed to bicarbonate or acetate dialysate. Our patient had normal serum bicarbonate levels. It is possible that lactate based dialysate may not be harmful during pregnancy and may provide an advantage. The relative inadequacy of PD in pregnancy is unlikely to be related to the presence of lactate in the dialysate.

The patient had preserved residual urine output of 300 mL per day despite being on dialysis for 3 years and this increased to over 1 L per day during pregnancy. We speculate that her tubular function remained intact throughout these years (her cause of ESRD being FSGS), and this may have resulted in preservation of aquaporin channels and principal cell function in the collecting tubules. Systemic arterial vasodilation occurs in the first trimester of pregnancy and this is coupled with stimulation of the renin-angiotensin-aldosterone system and hypotonicity. Arterial underfilling induces nonosmotic stimulation of arginine vasopressin and upregulation of aquaporin 2 followed by trafficking of this water channel to the apical membrane of principal cells along the collecting ducts [9]. In middle and late pregnancy there is also a significant increase in vasopressinase which is produced from placental trophoblasts, which enhances the clearance of vasopressin. A transient diabetes insipidus may ensue from this vasopressinase-mediated degradation of the vasopressin molecule. These mechanisms may explain why her urine output increased. We did not see a classic polyuric state due to diminished renal function from ESRD.

Our patient had a preterm delivery. A recent metaregression analysis [10] reveals that the risk of preterm delivery, early preterm delivery, and very early preterm delivery is approximately 80% in this population. The analysis showed a relationship between hours of dialysis per week in HD and preterm delivery and was significant for preterm deliveries and for small gestational age (SGA). SGA was closely associated with the number of dialysis sessions per week. Case report analysis suggested a lower incidence of SGA with HD versus PD (31% versus 66.7%, *p* = 0.015). The fact that increasing sessions of dialysis per week and increasing dialysis time decrease risk for preterm delivery and SGA suggests that middle molecule clearance may play a significant role in pregnancy outcomes. Our patient did not have SGA, and we speculate that this could be from increased middle molecule clearance from increased residual renal function and increased dialysis time. In concordance with data that has emerged since our patient's pregnancy, switching her to nocturnal hemodialysis with greater than 35 hours of hemodialysis per week may have provided her with the best outcome possible. However, she was unamenable to this change.

Growing literature supports intensive hemodialysis (>37 hours/week) [3] as the ideal mode of RRT in pregnancy due to the benefits mentioned previously. However, the patients in Hladunewich et al.'s study were on CHD or NHD, which does not equate with our patient who was receiving low-volume dialysate renal replacement therapy, and it is possible that patients on NxStage may benefit from even longer therapy times. Data for peritoneal dialysis in pregnancy reveals that the incidence of pregnancy is lower in PD patients (1.06 in PD versus 2.54 in HD). The live birth rate was also lower in PD (0.66 compared to 1.55 in HD) [11]. The theoretical concept of clearance with low-volume dialysate in PD is similar to NxStage system so we can speculate that pregnancy outcomes with the NxStage machine may be more comparable to PD. Hemodiafiltration has also been studied with promising outcomes as all pregnant patients had live births with gestational age greater than 30 weeks [12]. Haase et al. used hemodiafiltration due to the excellent cardiovascular tolerance HDF provides as well as the improved clearance of large solutes like phosphate while preserving high clearance for small solutes such as urea and creatinine. They achieved a weekly Kt/Vdp of 9.6. One disadvantage of this modality is the increased length of hospitalization. All patients had minimum hospitalization of 45 days until delivery.

Overall, pregnancy outcomes in women of childbearing age have improved tremendously in the last 30 years. It is imperative to discuss issues of fertility, conception, and pregnancy outcomes with differing dialysis modalities with female patients of childbearing age with advanced CKD or ESRD. We believe the NxStage System One machine deserves separate study from nocturnal or short daily dialysis in pregnant ESRD patients due to its distinctive method of using low-volume lactate-based dialysate.

## 4. Conclusions

The NxStage System One may be a beneficial renal replacement modality to obtain successful pregnancy outcomes in ESRD women of childbearing age and warrants further study.

## Figures and Tables

**Table 1:** 

	CHD	SDHD	NHD	NxStage HD
Treatments/wk	3	6	5-6	6
Treatment time	4	2-3	6–8	2.5–3.5
Blood flow rate (mL/min)	400	400	200	400
Dialysate flow rate (mL/min)	500	800	300	130
Single-pool Kt/V	1.2	0.5	1.8	0.5 (a)

CHD: conventional hemodialysis; SDHD: short daily hemodialysis; NHD: nocturnal hemodialysis; (a): using NxStage short daily prescription.

**Table 2:** Laboratory data.

Dates	Month 1	Month 2	Month 3	Month 4	Month 5	Month 6	Month 7	Month 8	Month 9
Post-HD weight (kg)	95.5	96.0	96.1	96.0	97.7	102.4	103.0	104.1	102.0
Hb (g/dL)	10.7	11.4	12.1	11.7	9.9	9.2	8.0	8.4	9.9
Iron sat. (%)	14	15	24	15	13	19	15	18	18
Calcium (mg/dL)	9.3	9.2	8.9	9.2	9.3	9.3	9.1	9.0	8.7
Phos. (mg/dL)	3.5	4.0	4.5	3.7	3.5	3.6	3.4	4.0	3.8
AST/ALT (U/L)	—	—	—	4/5	4/8	11/28	7/9	6/12	16/64
iPTH	398	544	645	702	576	314	360	336	453
Albumin (g/dL)	4.3	4.5	4.5	4.1	3.9	3.5	3.5	3.3	3.4
Bicarb. (mEq/L)	27	26	24	20	22	21	26	24	25
Avg. Pre-HD BUN (mg/dL)	50	49	46	44	30	29	28	29	29
Hours per week on HD	10	12	12	21	21	21	23	26	26
Dialysate vol./day (L)	30	30	30	30	40	40	40	45	45
Filtration fraction (%)	40	35	35	35	40	40	40	40	40
Avg. weekly Std. Kt/V	2.54	2.59	2.54	2.06	2.97	3.29	3.20	3.24	3.91

## References

[1] Hladunewich M., Hercz A. E., Keunen J., Chan C., Pierratos A. (2011). Pregnancy in end stage renal disease. *Seminars in Dialysis*.

[2] Piccoli G. B., Conijn A., Consiglio V. (2010). Pregnancy in dialysis patients: is the evidence strong enough to lead us to change our counseling policy?. *Clinical Journal of the American Society of Nephrology*.

[3] Hladunewich M. A., Hou S., Odutayo A. (2014). Intensive hemodialysis associates with improved pregnancy outcomes: a Canadian and United States cohort comparison. *Journal of the American Society of Nephrology*.

[4] Lockridge R. S., Pipkin M. (2008). Short and long nightly hemodialysis in the United States. *Hemodialysis International*.

[5] Bagon J. A., Vernaeve H., De Muylder X., Lafontaine J.-J., Martens J., Van Roost G. (1998). Pregnancy and dialysis. *American Journal of Kidney Diseases*.

[6] Kohn O. F., Coe F. L., Ing T. S. (2010). Solute kinetics with short-daily home hemodialysis using slow dialysate flow rate. *Hemodialysis International*.

[7] Weinhandl E. D., Gilbertson D. T., Collins A. J. (2016). Mortality, hospitalization, and technique failure in daily home hemodialysis and matched peritoneal dialysis patients: a Matched Cohort study. *American Journal of Kidney Diseases*.

[8] USRDS data http://www.usrds.org/2014/view/v2_01.aspx.

[9] Schrier R. W. (2010). Systemic arterial vasodilation, vasopressin, and vasopressinase in pregnancy. *Journal of the American Society of Nephrology*.

[10] Piccoli G. B., Minelli F., Versino E. (2015). Pregnancy in dialysis patients in the new millennium: a systematic review and meta-regression analysis correlating dialysis schedules and pregnancy outcomes. *Nephrology Dialysis Transplantation*.

[11] Shahir A. K., Briggs N., Katsoulis J., Levidiotis V. (2013). An observational outcomes study from 1966–2008, examining pregnancy and neonatal outcomes from dialysed women using data from the ANZDATA registry. *Nephrology*.

[12] Haase M., Morgera S., Bamberg C. (2005). A systematic approach to managing pregnant dialysis patients—the importance of an intensified haemodiafiltration protocol. *Nephrology Dialysis Transplantation*.

